# Discovery of novel PARP1/NRP1 dual-targeting inhibitors with strong antitumor potency

**DOI:** 10.3389/fphar.2024.1454957

**Published:** 2024-11-29

**Authors:** Juanjuan Liu, Yifei Geng, Su Jiang, Lixia Guan, Junyi Gao, Miao-Miao Niu, Jindong Li

**Affiliations:** ^1^ Department of Pharmacy, Taizhou School of Clinical Medicine, The Affiliated Taizhou People’s Hospital of Nanjing Medical University, Taizhou, China; ^2^ Department of Pharmaceutical Analysis, China Pharmaceutical University, Nanjing, China

**Keywords:** poly (ADP-ribose) polymerase-1 (PARP1), neuropilin-1 (NRP1), breast cancer, dual-targeting inhibitors, structure-based virtual screening

## Abstract

Given that overexpression of Poly (ADP-ribose) polymerase-1 (PARP1) and Neuropilin-1 (NRP1) is implicated in the pathogenesis of human breast cancer, the design of dual PARP1/NRP1 inhibitors has wide therapeutic prospect. However, there have been no reports of such inhibitors so far. Herein, we discovered novel small molecule inhibitors that simultaneously target PARP1 and NRP1 using structure-based virtual screening for the treatment of breast cancer. Notably, PPNR-4 was the most potent inhibitor targeting PARP1 (IC_50_ = 7.71 ± 0.39 nM) and NRP1 (IC_50_ = 24.48 ± 2.16 nM). PPNR-4 showed high affinity and binding stability to PARP1 and NRP1. The cytotoxicity assays showed that PPNR-4 demonstrated significant antiproliferative activity on MDA-MB-231 cells (IC_50_ = 0.21 μM) without effect on normal human cells. *In vivo* experiments exhibited that PPNR-4 showed more effective than the positive controls in inhibiting the growth of tumors. Overall, these data suggest that PPNR-4 is an effective antitumor candidate and deserves further research.

## 1 Introduction

Cancer, as a complex multifactorial disease that poses a major threat to human health, requires multiple therapeutic interventions (Ge et al., 2022). Worldwide, breast cancer (BC) is the most common cause of cancer in women (Łukasiewicz et al., 2021). Globally, more than 1.5 million women suffer from breast cancer annually (about a quarter of the women with cancer worldwide) (Sun et al., 2017). In particular, triple-negative breast cancer (TNBC) is the most malignant subtype, and patients typically have high rates of recurrence and low survival rates (Medina et al., 2020). TNBC is characterized by the absence of estrogen receptor, progesterone receptor and HER2 expression (Yin et al., 2020). To date, treatment options for TNBC are limited to highly toxic chemotherapy and systemic radiotherapy; however, the insensitivity of TNBC to radiotherapy has resulted in a poor 5-year prognosis (Geenen et al., 2018; Garrido-Castro et al., 2019; Zhang et al., 2023). Furthermore, the available targeted therapies did not significantly improve the survival of TNBC patients (Garrido-Castro et al., 2019). Thus, the clinical treatment of TNBC still faces great difficulties, and it is urgent to find new targeted therapeutic strategies and provide new drug design approaches (Collignon et al., 2016).

**FIGURE 1:**
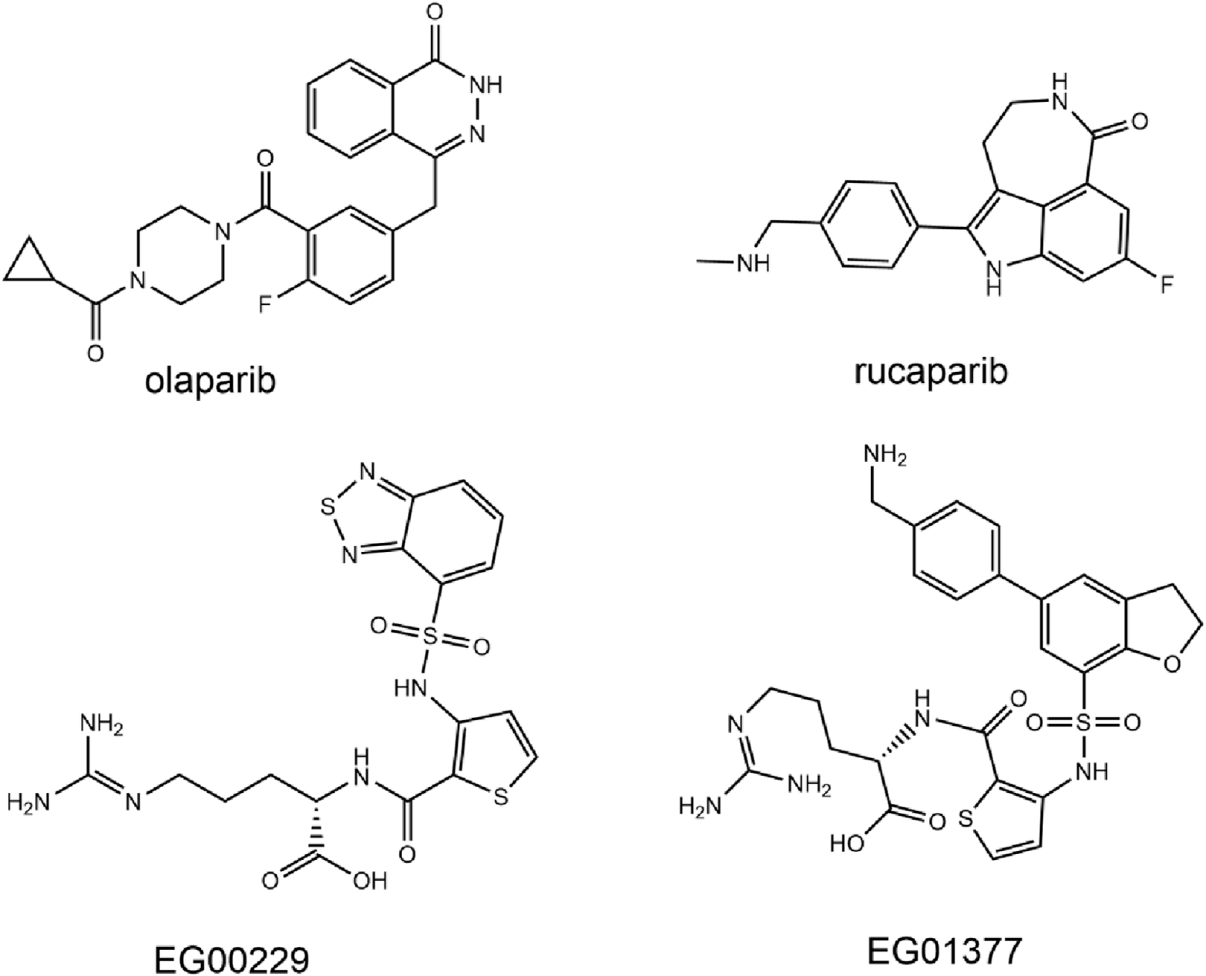
Reported PARP1 and NRP1 inhibitors.

Poly (ADP-ribose) polymerase-1 (PARP1) is a class of nuclear enzymes involved in DNA damage repair. PARP1 possesses an NAD-dependent catalytic activity and catalyzes the poly (ADP-ribose) covalently binding to nucleoprotein to repair damaged DNA (Krishnakumar and Kraus, 2010; Huang and Kraus, 2022). In the process of DNA damage repair (DDR), DNA double-strand breaks (DSBs) are mainly repaired through homologous recombination repair (HRR) pathways, and the dominant gene BRCA1/2 plays an important role in HRR (Gibson and Kraus, 2012; Luo J. et al., 2016; Gong et al., 2023). Approximately 10%–40% of TNBC patients have BRCA1/2 gene mutations (Chopra et al., 2020). In BRCA1 mutant breast cancer, the HRR pathway is damaged, and inhibition of PARP in cells leads to DNA damage accumulation and ultimately induces apoptosis (Helleday et al., 2005). Studies indicate that PARP1 inhibition leads to synthetic lethality in BRCA1 and BRCA2-deficient cell lines (Cong et al., 2021). In addition, PARP1 expression is upregulated in about 70% of primary breast cancers, especially TNBC (Ossovskaya et al., 2010). Given its potential biological role in malignant tumors, PARP1 has emerged as a promising target for anticancer drugs. In recent years, several PARP1 inhibitors such as olaparib (Figure 1), rucaparib, niraparib and talazoparib have been approved for the clinical treatment of BRCA-mutant advanced ovarian cancer and metastatic breast cancer (Zhao et al., 2020). However, resistance due to mechanisms such as BRCA gene mutation reversal reduces the clinical efficacy of PARP inhibitors, and HR-proficient cancers are not sensitive to PARP1 inhibitors (Lord and Ashworth, 2013; Lord and Ashworth, 2017; Ge et al., 2022).

Neuropilin-1 (NRP1) is a multifunctional, transmembrane, non-tyrosine kinase surface glycoprotein (Roy et al., 2017). Moreover, NRP1 is a co-receptor for vascular endothelial growth factor (VEGF), and blocking NRP1 can inhibit tumor growth by suppressing angiogenesis (Zhao et al., 2021a). It has been shown that NRP1 is expressed in breast cancer spheroid cells and that the VEGF-A/NRP1 axis activates the Wnt/β-catenin pathway in breast cancer cells (Zhang et al., 2017). In addition, NRP1 is expressed at higher levels in TNBC cells than in non-TNBC cells and contributes to the proliferation and metastasis of TNBC cells (Zhang et al., 2021). Knockdown of NRP1 inhibits proliferation, migration and invasion, and promotes apoptosis in MDA-MB-231 cells (Luo M. et al., 2016). Therefore, NRP1 inhibitors may be a promising antitumor angiogenesis drug to prevent cancer metastasis. While a number of peptides targeting NRP1 and mAb antagonizing NRP1 signaling have been reported, they still have weak therapeutic effects on solid tumors (Patnaik et al., 2014; Weekes et al., 2014; Kamarulzaman et al., 2017; Zhao et al., 2021b). Developing a potent small molecule NRP1 antagonist with improved *in vivo* efficacy would be attractive (Powell et al., 2018). For example, the small molecule drugs EG00229 and EG01377 (Figure 1) found by Selwood et al., showed rather potent activities in the lower micromolar range (Jarvis et al., 2010; Powell et al., 2018).

Despite important advances in cancer treatment with PARP1 inhibitors, HR-proficient cancers are resistant to PARP1 inhibitors (Heeke et al., 2018; Ge et al., 2022). Thus, PARP1 inhibitors are still ineffective in the treatment of some cancer patients (Goel et al., 2021). Inducing HR-deficient or BRCAness phenotypes in tumor cells by drugs would be an ideal strategy to overcome drug resistance (Han et al., 2020; Klotz and Wimberger, 2020). PARP1 inhibitors in combination with other drugs such as EGFR inhibitors and CDK12 inhibitors can increase the sensitivity of tumor cells to PARP1 inhibitors by inducing HR-deficient (Liu et al., 2014; Johnson et al., 2016; He et al., 2024). Encouragingly, Vescarelli et al. found that NRP1 is expressed at high levels in drug-resistant cells and is upregulated in partially sensitized cells (UWB-BRCA) after prolonged olaparib treatment. Selective inhibition of NRP1 can overcome olaparib resistance in drug-resistant cells (Vescarelli et al., 2020). Therefore, targeted inhibition of NRP1 restores the sensitivity of drug-resistant cells to PARPi. There findings support the importance of targeting PARP1 and NRP1 simultaneously. On the other hand, PARP inhibitors present antiangiogenic activity both *in vitro* and *in vivo*, which may exert synergistic therapeutic effects with angiogenesis inhibitors such as NRP1 inhibitors (Tentori et al., 2007). The use of angiogenesis inhibitors such as NRP1i in combination with other treatments has been approved by the FDA. Martí et al. also concluded that the combination of angiogenesis and PARP inhibitors will be likely safe (Martí et al., 2020). Therefore, we aimed to simultaneously target PARP1 and NRP1, which may be highly attractive and promising. To the best of our knowledge, there are no reports on dual-targeting inhibitors of PARP1 and NRP1.

Structure-based virtual screening approach is a convenient and powerful tool in the early stages of drug discovery (Lionta et al., 2014; He et al., 2024). Screening active compounds from virtual compound libraries involves utilizing key structural and physicochemical properties of ligands and targets (Vazquez et al., 2020). The combined strategy of pharmacophore modeling and molecular docking enables efficient and accurate identification of lead compounds from large databases (Bajusz and Keserű, 2022). Our previous study successfully identified a series of dual-targeted drugs through virtual screening (Zheng et al., 2021; Zhou et al., 2022). In this study, we reported a novel PARP1/NRP1 dual-targeting inhibitor (PPNR-4) through structure-based virtual screening. PPNR-4 showed high inhibitory effects with both PARP1 and NRP1. The binding affinity and stability of PPNR-4 were further determined by MST experiments and MD simulations. Both *in vitro* and *in vivo* experiments confirmed the antitumor effects of PPNR-4. In conclusion, this work identified the first PARP1/NRP1 dual-targeted inhibitor and provided an effective strategy for constructing dual-targeted drugs for efficient tumor therapy.

## 2 Materials and methods

### 2.1 Cell culture and materials

The breast cancer cell lines (MDA-MB-231) and normal human cell lines (MCF-10A, L02, FHC, PNT1A, HEL-299, and HEK-293) were purchased from The American Type Culture Collection (ATCC) (Manassas, VA, United States). The cells were cultured with Roswell Park Memorial Institute (RPMI)-1,640 medium supplemented with 10% (v/v) fetal bovine serum, 50 units/mL of penicillin, and 50 μg/mL of streptomycin. The cell culture system was maintained at 37°C in a humidified atmosphere containing 5% CO_2_. Hit compounds were purchased from WuXi AppTec. Recombinant human PARP1 (Accession No. NM_001618) and NRP1 (Accession No. O14786-2) proteins were purchased from Abcam (Cambridge, MA, United States).

### 2.2 Pharmacophore construction

The crystal structures of PARP1 (PDB ID: 7KK4) and NRP1 (PDB ID: 3I97) proteins in complex with ligands were obtained from the Protein Data Bank (PDB), and were imported into the Molecular Operating Environment (MOE, Chemical Computing Group Inc, Montreal, Quebec, Canada), respectively. Firstly, the above crystal structures were optimized using the QuickPrep tool of MOE, including removal of unbound water, calculation of partial charges, addition of polar hydrogen, and energy minimization. Then, the Ligand Interactions tool in MOE was used to analyze the interaction relationship between the protein and ligand of PARP1. Based on the analysis of the above interactions, pharmacophore models were constructed to include hydrogen bond acceptors, hydrogen bond donors, and aromatic centers.

### 2.3 Virtual screening

A database containing 53,357 compounds was created using combinatorial chemistry methods. Then the energy minimization program of MOE was used to convert the 2D structures of all compounds into 3D structures. The pharmacophore models of PARP1 previously constructed were used to perform virtual screening in the database. Next, the screened compounds were further subjected to molecular docking based on the above crystal structures of PARP1 and NRP1 in MOE. Each compound was docked to the active sites of PARP1 and NRP1 using the Dock tool of MOE. The docking results were evaluated using the Triangle Matcher method and the London dG scoring algorithm. Lower docking score indicates stronger binding affinity.

### 2.4 *In vitro* PARP1 inhibition assay

The method was carried out as described previously (Wang et al., 2021). In each well, 50 μL of 1 × PARP1 buffer was added and incubated for 30 min at room temperature. The 1 × PARP1 buffer was removed from the wells, and then 25 μL of 1 × PARP1 cocktail was added to each well along with varying doses of compounds and PARP1 enzyme (0.6 units/well). Incubated and washed twice with PBS and 0.1% Triton X-100. Then added 50 μL Strep-HRP and washed twice. Equal volumes (100 μL) of PeroxyGlow A and B were added to each well and immediately take chemiluminescent readings. Data were analyzed by GraphPad Prism 9 software based on chemiluminescence readings of chemiluminescent substrates of Strep-HRP in each well. The IC_50_ value was were calculated by nonlinear curve fitting in GraphPad Prism 9.

### 2.5 *In vitro* NRP1 inhibition assay

The method was performed as described previously (Jarvis et al., 2010). Radiolabeled binding displacement experiments were performed as described previously (Jia et al., 2006). Confluent adenovirus NRP1-transfected HUVEC cells in 96-well plates were washed twice with PBS. At 4°C various concentrations of compounds diluted in binding medium (Dulbecco’s modified Eagle’s medium, 25 mM HEPES, pH 7.3, containing 0.1% BSA) were added, followed by addition of the indicated concentration of ^125^I-VEGF-A (1,200–1800 Ci/mmol, GE Healthcare, United Kingdom). After 2 h of incubation at 4°C, the medium was aspirated, and washed four times with cold phosphate-buffered saline. The cells were lysed with 0.25 M NaOH, 0.5% SDS solution, and the bound radioactivity of the lysates was measured. IC_50_ values were calculated from the competition curves of the compounds in the ^125^I-VEGF-A165 binding assay using GraphPad Prism 9 software. Using log(inhibitors) vs response-Variable slope equation in the GraphPad Prism 9 to fit the curves. The equation was as follows: (Top and Bottom are the responses, respectively, at the top and bottom of the curve, Y is the inhibitory response at a given compound concentration X, and the Hillslope describes the steepness of the curve.)

$$Y=\text{Bottom}+\frac{\left(\text{Top}-\text{Bottom}\right)}{1+10^{\left(\log{\text{IC}}_{50}-x\right)\text{Hillslope}}}$$

### 2.6 Selectivity profile

The selectivity profile of PPNR-4 was performed by SelectScreen Enzyme Profiling Service (Thermo Fisher Scientific).

### 2.7 Microscale thermophoresis (MST) assay

To evaluate the binding affinity of test compounds and proteins, an MST assay was performed using Monolith NT. Automated (NanoTemper Technologies), as described previously (Yin et al., 2022). The recombinant PARP1 or NRP1 protein was labeled using the Lys labeling kit RED-NHS second Generation (MO-L011, NanoTemper) according to the manufacturer’s instructions. The final labeled protein concentration was 50 nM, which was mixed with different concentrations of test compounds by multiple pipetting. All samples were diluted in 1 × PBST. MST analysis was performed using the Monolith NT. Automated (NanoTemper Technologies), and *K*_d_ values was measured using the MO. Affinity Analysis software.

### 2.8 Molecular dynamics (MD) simulations

The crystal structures of PARP1 (PDB ID: 7KK4) and NRP1 (PDB ID: 3I97) were downloaded from the PDB. The molecular dynamics (MD) simulations were performed using GROMACS (version 2022) under the AMBER99SB-ILDN force field. PPNR-4 was imported into the Acpype Server (www.bio2byte.be) to obtain the topology file. The system was dissolved by an SPC/E water model in a 1.0 nm cubic box. The water molecules were supplanted by sodium ions (Na^+^) and chloride ions (Cl^−^) to keep the complex system neutral. Subsequently, the steepest descent algorithm with 5,000 steps was used to minimize the system energy. The V-rescale thermostat was used to sustain the system temperature at 300 K during the 100 ps NVT simulation. After that, NPT simulation was further conducted for 100 ps using a Parinello-Rahman barostat to maintain the system pressure at 1 bar. Finally, a 50 ns MD simulation was implemented and trajectory data was recorded at 10 ps intervals. The binding free energy was calculated using the MMGBSA method, and 400 frames from a 50 ns trajectory were extracted for analysis. The data were processed using GraphPad Prism 9 software.

### 2.9 *In vitro* antiproliferation assay

Cell viability was measured by MTT assays. MDA-MB-231, MCF-10A, L02, FHC, PNT1A, HEL-299, and HEK-293 cell lines were seeded in 96-well plates at 5 ×10^4^ cells/well and incubated overnight. Different concentrations of PPNR1-5 or positive control were then added to each well and cultured at 37°C for 72 h. Afterward, the medium was removed. MTT (5 mg/mL) was dissolved in phosphate-buffered saline (PBS) and filter-sterilized, and then, 20 μL of the prepared MTT solution was added to each well, and the cells were incubated for another 4 h until a purple precipitate was visible. The supernatant was removed by centrifugation, and DMSO (150 μL/well) was added to dissolve the insoluble crystals. The absorbance was determined using an enzyme-linked immunosorbent assay reader at a test wavelength of 570 nm. The assays were performed three times in a parallel manner. The non-linear regression was used to plot a Dose-Response-Inhibition to determine the value of the IC_50_. The cell inhibition rate was calculated at 2 μM concentration of compound treatment. The data were analyzed using GraphPad Prism 9 software.

### 2.10 RNA isolation and quantitative real time polymerase chain reaction

The method was performed as reported previously (Zare et al., 2019). Briefly, total RNA was extracted from the samples using RNX-Plus solution, and complementary DNA to the total RNA was synthesized using the RevertAid First Strand cDNA Synthesis Kit and gradient thermal cycler. 1 μL of the cDNA samples was added to the qRT-PCR master mix. The 48-well plate containing all reagents was briefly centrifuged and analyzed on an ABI Step One Real-Time PCR system (AppliedBiosystems, ABI, United States).

### 2.11 Tube formation analysis

The effect of PPNR-4 on the formation of vascular-like structures in HUVECs was determined as previously reported (Carpentier et al., 2020). 50 μL of thawed Matrigel (#356234, BD Biosciences, United States) was added to a 96-well plate and incubated at 37°C for 30 min. After Matrigel gelation, HUVECs (1 × 10^5^ cells/mL) were seeded into the basement membrane matrix. Each well was filled with 100 μL of cell suspension. Then, different concentrations of PPNR-4 (0, 2, 6, and 18 μM) were added and the cultures were incubated for 8 h (37°C, 5% CO_2_). Finally, five randomly selected fields per well were photographed with an inverted light microscope. The results were analyzed by ImageJ software to measure and record the number of branch points and tube length for each field. The specific steps are to download the “angiogenesis analyzer” plugin and click on ImageJ, Plugings, Macros, Install, angiogenesis analyzer in sequence. At least three parallel replicates were performed for each condition. The relative number of tubes formation was analyzed using GraphPad Prism 9 software.

### 2.12 *In vivo* pharmacokinetic studies

PPNR-4 was administered (10 mg/kg) *via* p. o. in SD rats. Blood samples (0.25 mL) were collected at 0.25, 0.5, 1, 2, 4, 8, 16, and 24 h after administration and centrifuged to obtain the plasma fraction. In a 96-well plate, the plasma sample (50 μL) was transferred, and the internal standard methanol/acetonitrile (1:1, v/v) solution (200 μL) was added. After vortexing for 5 min, the mixture was centrifuged (12,000 rpm, 5 min) and the supernatant was obtained (60.0 μL). Liquid chromatography and tandem mass spectrometry (LC-MS/MS) system was used to study pharmacokinetics. The data were processed by Phoenix.

### 2.13 *In vivo* antitumor assay

Twenty-four female BALB/c nude mice (body weight 16–20 g) at 4–6 weeks old were purchased from Changzhou Cavens Experimental Animal Limited Company (Changzhou, China). Mice were adaptively housed for 1 week in a specific pathogen-free (SPF) environment with a temperature of 25°C ± 2°C, a 12-hour light/dark cycle, and free access to food and water. MDA-MB-231 breast cancer cells (200 μL, 1×10^7^ cells) were suspended in PBS to formulate cell suspension, and 0.1 mL of cells were injected into the axilla of the left forelimb of mice by subcutaneous injection. Tumor growth was observed in mice. When the tumor size reached an average of 90–120 mm^3^, the mice were randomly divided into four groups (6 mice per group), and each group was injected intraperitoneally with 5 mg/kg vehicle, 5 mg/kg EG00229, 5 mg/kg olaparib, and 5 mg/kg PPNR-4. Equal concentrations of drug-free PBS buffer were used for the vehicle group. Each group was administered every 3 days and the body weight and tumor size of the mice were recorded. Mice were executed when the tumor volume approached 2000 mm^3^. Tumor volumes of all mice were measured using vernier calipers. The mice were placed belly up and the longest diameter of the tumor was measured with the inner bayonet of a vernier caliper, and the shortest transverse diameter perpendicular to the diameter was measured with the inner bayonet of a vernier caliper and recorded. Tumor volume was estimated according to the following formula: (c × c × d)/2 (c, the smallest diameter; d, the largest diameter). All animal experiments were performed and approved by the Ethics Committee of China Pharmaceutical University.

## 3 Results and discussion

### 3.1 Structure-based pharmacophore modelling

**FIGURE 2:**
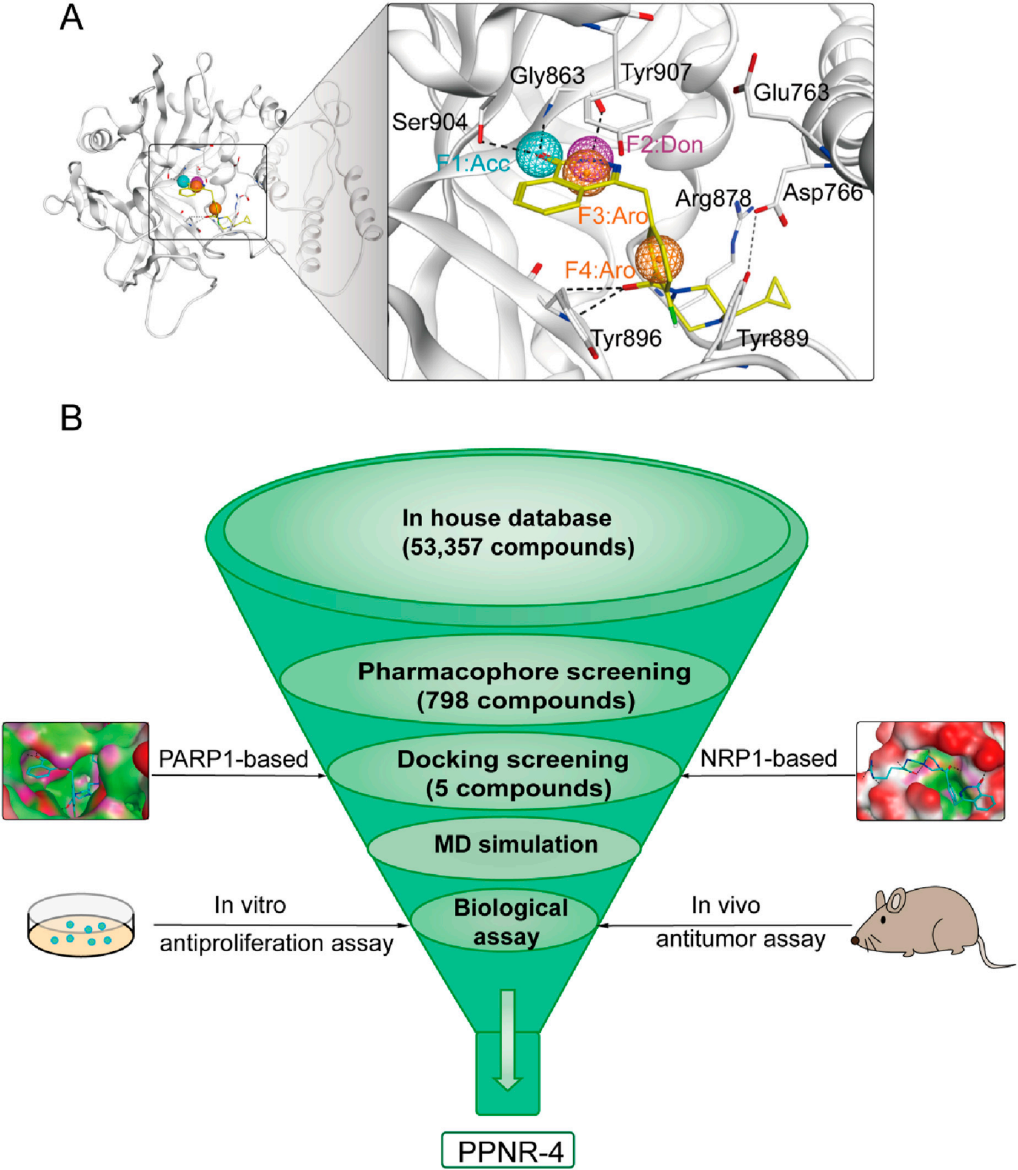
**(A)** The pharmacophore model of PARP1. The residues in active site of PARP1 were presented as sticks with atoms (carbon: gray, oxygen: red, and nitrogen: blue). **(B)** The workflow of multi-step virtual screening of dual-targeting PARP1/NRP1 inhibitors.

Pharmacophore modeling describes the three-dimensional (3D) chemical and structural properties that ligands must have to ensure the optimal ligand-receptor binding mode (Meyer et al., 2012). To enhance the identification process of new dual-targeted drugs, we obtained a high-resolution crystal structure of PARP1 in complex with the original ligand (PDB ID: 7KK4) and generated a PARP1-based pharmacophore model using the Pharmacophore Query Editor of the MOE. The ligand served as a hydrogen bond acceptor to form hydrogen bonds with Ser904 and Gly863 residues. Meanwhile, the ligand served as a hydrogen bond donor to form an additional hydrogen bond with Gly863. The ligand served as an aromatic center to create π-π interactions with Tyr907, Tyr889, and Tyr896 residues. The final pharmacophore model contained four pharmacophore features: a hydrogen-bond acceptor feature (F1: Acc, cyan color), a hydrogen-bond donor feature (F2: Don, purple color), two aromatic center features (F3 and F4: Aro, orange color). As shown in Figure 2A, features of the pharmacophore model reflected key interaction points for ligand binding to PARP1: i) the Acc feature (F1) corresponds to residues Ser904 and Gly863; ii) the Don feature (F2) corresponds to Gly863; iii) both the Aro features (F3 and F4) correspond to residues Tyr907, Tyr889, and Tyr896.

### 3.2 Virtual screening

**FIGURE 3:**
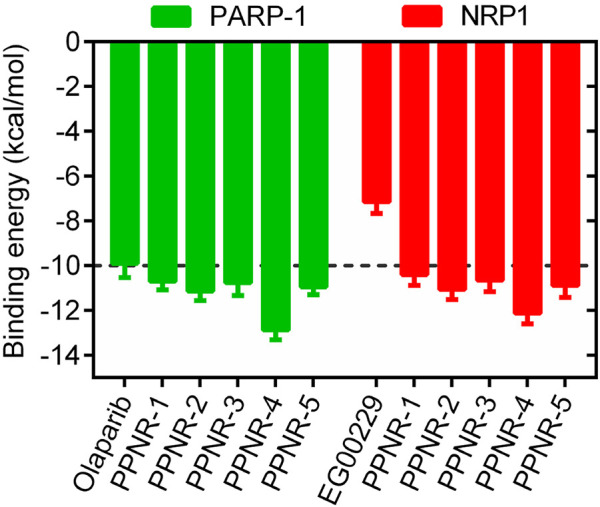
The binding free energy (kcal/mol) of five hit compounds (PPNR 1–5).

**FIGURE 4:**
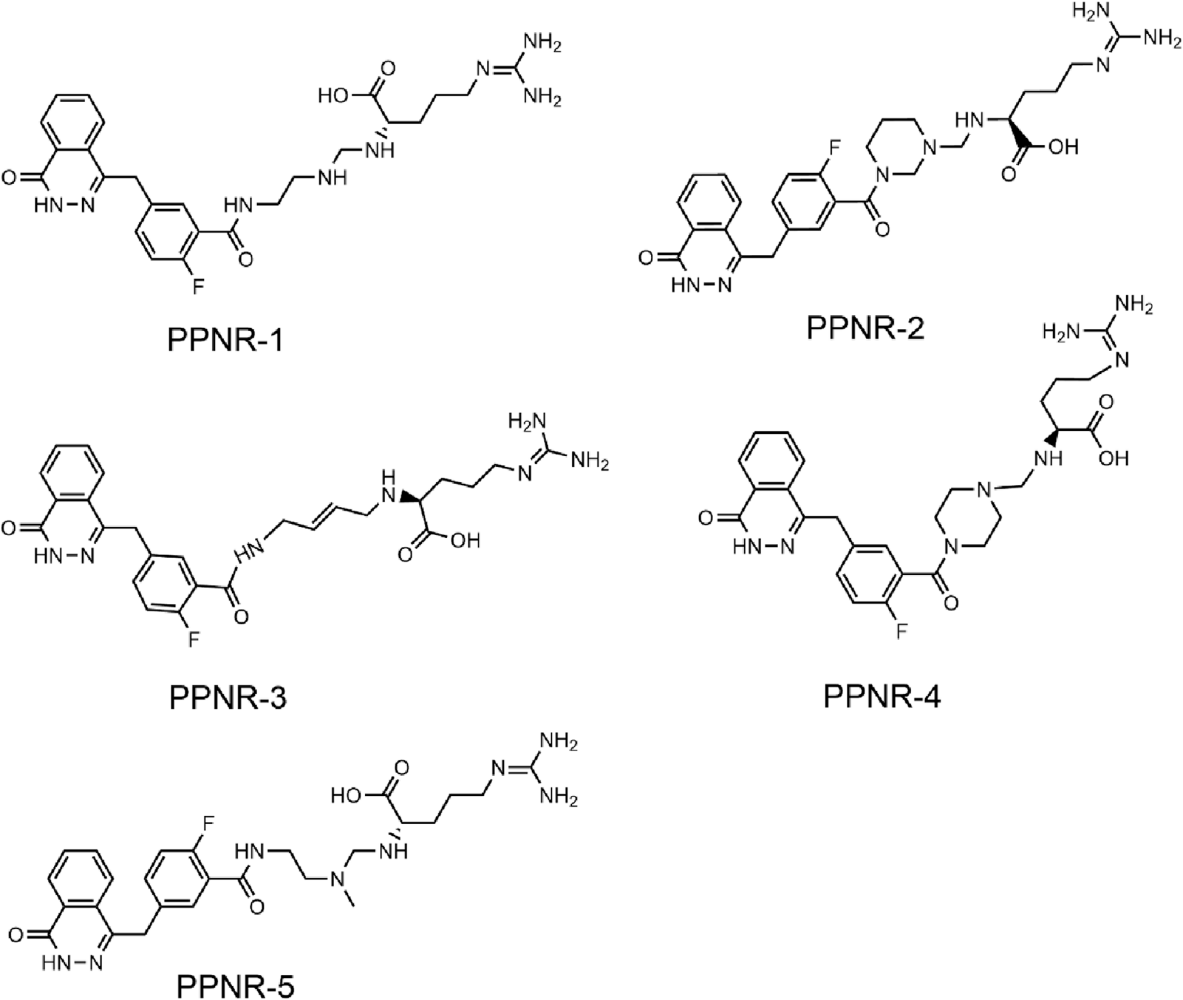
The chemical structures of PPNR 1-5.

The multistep virtual screening process of this study is shown in Figure 2B. Novel dual-targeted inhibitors of PARP1 and NRP1 were identified from the constructed compound database through the integrated virtual screening. High-resolution crystal structures of PARP1 (PDB ID: 7KK4) and NRP1 (PDB ID: 3I97) were downloaded from the PDB. First, the in-house two-dimensional structural database of 53,357 compounds originating from the CPU laboratory was transformed into a three-dimensional structure by energy minimization. The pharmacophore features created above were used as a 3D search query for compounds with strong binding energy retrieved from the database using MOE. Smaller RMSD values represent better shape fit and interaction between ligand and receptor, which indicates higher affinity and inhibitory activity (Aboul-Fadl et al., 2010). The 798 compounds obtained from the pharmacophore screening were subsequently screened for molecular docking. Docking scores were used to evaluate the binding affinity of compounds to PARP1 and NRP1, with lower scores indicating higher binding affinity. First, compounds in the database were docked to the active site of PARP1 to screen for PARP1-targeted compounds, and docking scores were calculated. We used olaparib, a previously reported PARP1 inhibitor, as a positive control. The docking score for olaparib was −9.89 kcal/mol. Thus, −10 kcal/mol was used as a threshold to select 37 compounds with docking scores below this threshold. Next, these 37 compounds were docked to NRP1. The NRP1 inhibitor, EG00229 served as a positive control. Due to the docking score of, EG00229 was −7.14 kcal/mol, we used −7.14 kcal/mol as the cutoff value and selected top-ranked compounds. Ultimately, the top five hits (termed as PPNR 1–5) that simultaneously satisfied the above docking cutoff values were selected for further affinity testing. Docking scores and structures of PPNR 1-5 are shown in Figures 3, 4, respectively.

### 3.3 Structure-activity relationship

**FIGURE 5:**
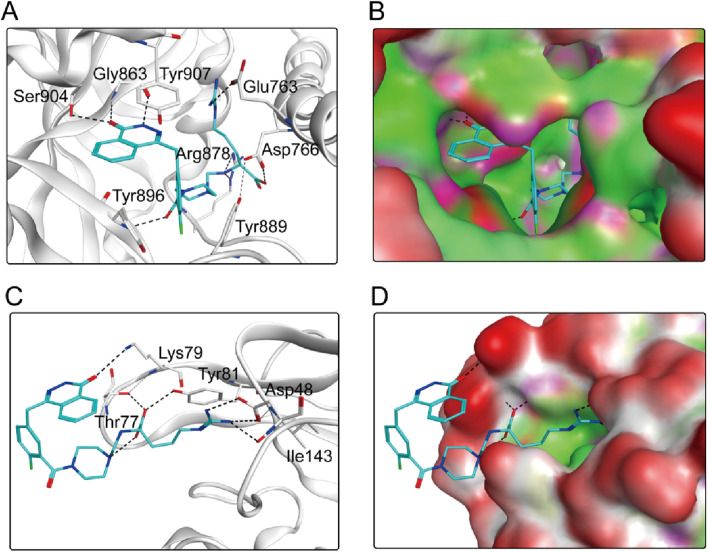
**(A, B)** The binding mode of PPNR-4 (cyan sticks) in the active site of PARP1. **(C, D)** The binding mode of PPNR-4 (cyan sticks) in the active site of NRP1. Residues in the active site are shown as grey sticks. The hydrogen bonds are represented in black dashed lines.

According to the docking results shown above, PPNR-4 had the lowest binding free energy in docking with PARP1 and NRP1. Based on the docking of PARP1 and NRP1, we further investigated how PPNR-4 interacts with the binding sites. Figures 5A, B show the binding mode and binding surface map of PPNR-4 docked to PARP1 protein. It is well established that the agents target the NAD binding site by occupying the nicotinamide pocket. PPNR-4 was stabilized at the PARP1 binding site by forming hydrogen bonds with the key residues Ser904, Gly863, Tyr896, Arg878, Asp766, and Glu763, which anchored the direction of the binding process. In addition, all of PPNR 1-5 contained 1-(2H)-phthalazinone moiety. The 1-(2H)-phthalazinone pharmacophore formed π-π stacking interactions with Tyr896 and Tyr907 of the PARP1 catalytic site, which was critical for inhibitor activity. As shown in the binding surface map, PPNR-4 nicely occupied the pocket of PARP1. Figures 5C, D reveal the binding mode and binding surface map of PPNR-4 docked to NRP1 protein. PPNR-4 matched well with the long and narrow binding pocket of NRP1. The guanidinium group of PPNR-4 was engaged in hydrophobic interactions with Asp48 and Ile143 residues on the outside of the pocket, which acted as a key force for the binding. Meanwhile, PPNR-4 formed additional hydrogen bonds with Lys79, Tyr81, and Thr77, anchoring it to the catalytic site of NRP1. Notably, we also found that compounds containing hexahydro N-heterocycle (PPNR-2 and PPNR-4) showed stronger binding than the others in PPNR 1–5. This suggests that the hexahydro N-heterocycle linker was beneficial to the dual PARP1/NRP1 inhibitory activities.

### 3.4 Inhibitory effect and binding affinity

We performed enzyme activity inhibition assays to evaluate the inhibitory effects of PPNR 1-5 on PARP1 and NRP1. As shown in Table 1, the screened compounds generally showed potent inhibitory activity against both targets. The PARP1 inhibitor olaparib and NRP1 inhibitor, EG00229 were used as positive controls. PPNR 1-5 showed excellent activity at the nanomolar level. Notably, PPNR-4 exhibited the most potent inhibitory effects. The IC_50_ values of PPNR-4 (PARP1 IC_50_ = 7.71 ± 0.39 nM, NRP1 IC_50_ = 24.48 ± 2.16 nM) were lower than that of olaparib (IC_50_ = 8.37 ± 1.64 nM) and, EG00229 (IC_50_ = 640.21 ± 34.73 nM) (Supplementary Figure S1). Additionally, PPNR-4 showed the lowest docking score and the strongest inhibitory activity, which was consistent with the results in molecular docking studies. To further verify the potential off-target effect of PPNR-4, we tested the inhibitory effects of PPNR-4 on other isoforms of PARP and NRP family. The results are shown in Table 2. PPNR-4 was found to have no significant inhibitory effect on all other isoforms (IC_50_ > 10 μM). In addition, we tested the inhibitory activity of PPNR-4 on 123 kinase panel. The results are shown in Table 3. PPNR-4 exhibited no significant inhibitory effects on any of the 123 kinases (IC_50_ > 10 μM). Thus, PARP1 and NRP1 are the primary targets of PPNR-4, suggesting that PPNR-4 has no potential off-target effects.

**TABLE 1:** Dual inhibitory effects of PPNR 1-5 on PARP1 and NRP1.

Compounds	PARP1 (IC_50_, nM)	NRP1 (IC_50_, nM)
PPNR-1	70.26 ± 3.13	94.08 ± 4.24
PPNR-2	29.32 ± 2.57	31.22 ± 3.95
PPNR-3	63.25 ± 4.02	72.86 ± 3.41
PPNR-4	7.71 ± 0.39	24.48 ± 2.16
PPNR-5	41.36 ± 2.86	57.13 ± 3.27
Olaparib	8.37 ± 1.64	No inhibition
EG00229	No inhibition	640.21 ± 34.73

**TABLE 2:** The inhibitory activity of PPNR-4 on other isoforms of PARP and NRP family.

Name	IC_50_ (μM)	Name	IC_50_ (μM)	Name	IC_50_ (μM)
PARP2	>10	PARP8	>10	PARP14	>10
PARP3	>10	PARP9	>10	PARP15	>10
PARP4	>10	PARP10	>10	PARP16	>10
PARP5	>10	PARP11	>10	PARP17	>10
PARP6	>10	PARP12	>10	NRP2	>10
PARP7	>10	PARP13	>10		

**TABLE 3:** The inhibitory activity of PPNR-4 on 123 kinase panel.

Name	IC_50_ (μM)	Name	IC_50_ (μM)	Name	IC_50_ (μM)
ABL1	>10	DDR2	>10	HIPK1	>10
ABL2	>10	DNA-PK	>10	HIPK2	>10
ACK	>10	DYRK1A	>10	HIPK3	>10
AKT1	>10	DYRK1B	>10	HIPK4	>10
AKT2	>10	DYRK2	>10	IGF1R	>10
AKT3	>10	DYRK3	>10	INSR	>10
ALK	>10	DYRK4	>10	ITK	>10
ALK4	>10	EGFR	>10	JAK1	>10
ALK6	>10	EPHA1	>10	JAK2	>10
AMPKα1/β1/γ1	>10	EPHA2	>10	JAK3	>10
AMPKα2/β1/γ1	>10	EPHA3	>10	JNK1	>10
AurA	>10	EPHA4	>10	JNK2	>10
AurB	>10	EPHA5	>10	JNK3	>10
AurC	>10	EPHA6	>10	KDR	>10
AXL	>10	EPHA7	>10	KHS	>10
BLK	>10	EPHA8	>10	KIT	>10
BMX	>10	EPHB1	>10	LCK	>10
BRAF	>10	EPHB2	>10	LOK	>10
BRK	>10	EPHB3	>10	LYNa	>10
BRSK1	>10	EPHB4	>10	MARK1	>10
BRSK2	>10	Erk1	>10	MARK2	>10
BTK	>10	Erk5	>10	MARK3	>10
CaMK1α	>10	Erk7	>10	MARK4	>10
CaMK2α	>10	FAK	>10	MER	>10
CLK1	>10	FER	>10	MET	>10
CLK3	>10	FES	>10	MINK	>10
CLK4	>10	FGFR1	>10	MLK1	>10
CRAF	>10	FGFR2	>10	MLK3	>10
CSK	>10	FGFR3	>10	MRCKα	>10
CHK1	>10	FGFR4	>10	MRCKβ	>10
CHK2	>10	FGR	>10	MSK1	>10
CK1α	>10	FLT1	>10	MST1	>10
CK1γ1	>10	FLT3	>10	MST2	>10
CK1γ2	>10	FLT4	>10	MST3	>10
CK1γ3	>10	CSF1R	>10	MUSK	>10
CK1δ	>10	FRK	>10	NEK1	>10
CK1ε	>10	GLK	>10	NEK2	>10
DAPK1	>10	GLK	>10	NEK9	>10
DCAMKL1	>10	GSK3α	>10	NIM1K	>10
DCAMKL2	>10	GSK3β	>10	NuaK1	>10
DDR1	>10	HCK	>10	NuaK2	>10

We performed MicroScale Thermophoresis (MST) assay to demonstrate the binding of PPNR 1-5 to the active sites of PARP1 and NRP1. The dissociation constants (*K*_d_) of the PPNR 1-5 to PARP1 and NRP1 measured by MST are shown in Table 4. The previously reported olaparib and, EG00229 were used as positive controls. The dissociation constants (*K*_d_) of olaparib and, EG00229 to PARP1 or NRP1 were 89.86 nM and 519.42 nM, respectively. The *K*_d_ values of PPNR 1-5 binding to PARP1 varied from 7.15 nM to 75.14 nM, and binding to NRP1 varied from 16.61 nM to 91.52 nM. As expected, PPNR-4 exhibited the highest affinity and specificity for PARP1 (*K*_d_ = 7.15 nM) and NRP1 (*K*_d_ = 16.61 nM) (Supplementary Figure S2). In addition, MST testing showed that PPNR-4 was not specific for other PARP and NRP family members and a few non-related proteins (Table 5).

**TABLE 4:** Affinity evaluation of PPNR 1-5 binding to PARP1 and NRP1.

Compounds	PARP1 (*K*_d_, nM)	NRP1 (*K*_d_, nM)
PPNR-1	75.14	91.52
PPNR-2	28.38	30.97
PPNR-3	61.07	71.29
PPNR-4	7.15	16.61
PPNR-5	39.97	55.28
Olaparib	89.86	no binding
EG00229	no binding	519.42

**TABLE 5:** Specificity testing of PPNR-4 on other PARP and NRP family members and a few non-related proteins by MST assay.

Name	*K*_d_ (μM)	Name	*K*_d_ (μM)
PARP2	>10	PARP13	>10
PARP3	>10	PARP14	>10
PARP4	>10	PARP15	>10
PARP5	>10	PARP16	>10
PARP6	>10	PARP17	>10
PARP7	>10	NRP2	>10
PARP8	>10	ATR	>10
PARP9	>10	BACA1	>10
PARP10	>10	BACH1	>10
PARP11	>10	CHEK2	>10
PARP12	>10	RAD51	>10

### 3.5 Molecular dynamics (MD) simulations

**FIGURE 6:**
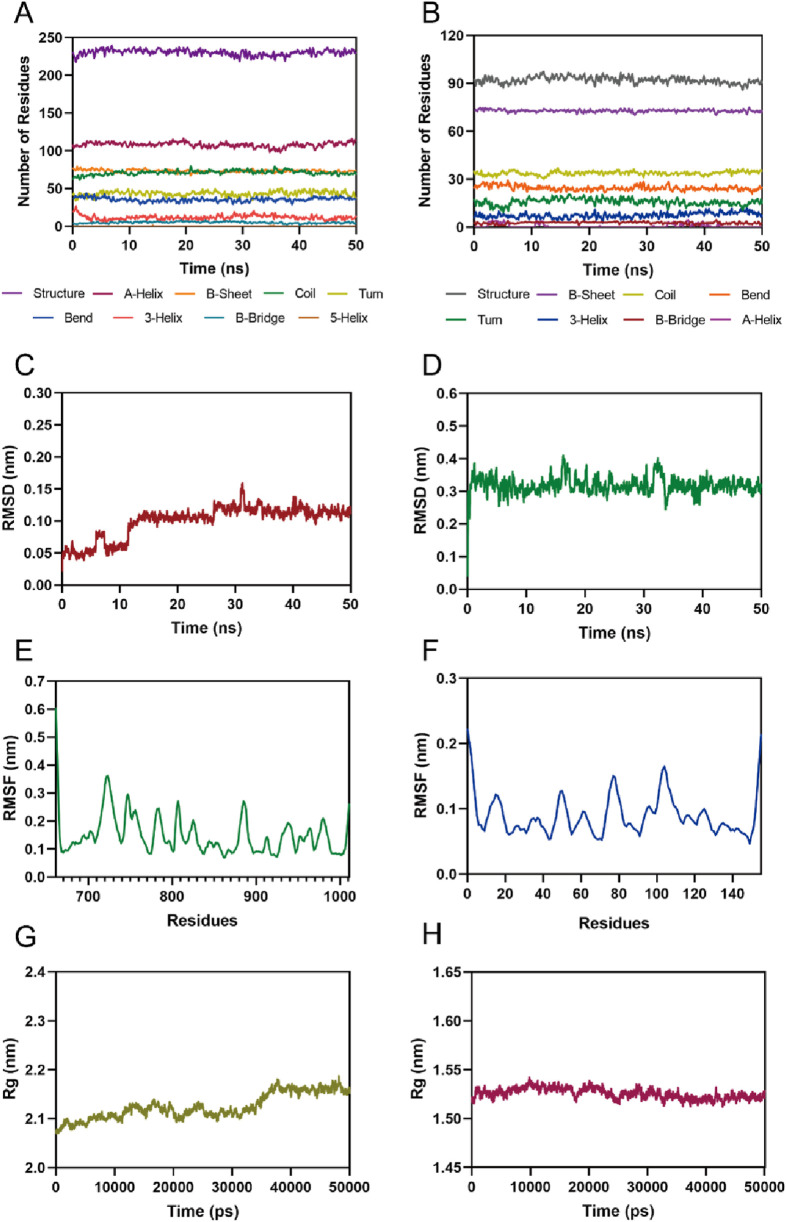
MD simulation of PPNR-4 in complex with PARP1 and NRP1. **(A, B)** The secondary structures analysis of PARP1 and NRP1, respectively. **(C)** The RMSD of the PPNR-4 in the PARP1-PPNR-4 complex. **(D)** The RMSD of the PPNR-4 in the NRP1-PPNR-4 complex. **(E)** The RMSF of Cα atoms of PARP1. **(F)** The RMSF of Cα atoms of NRP1. **(G)** Radius of gyration (Rg) of PARP1. **(H)** Radius of gyration (Rg) of NRP1.

Given the excellent inhibitory activity of PPNR-4, we further assessed the stability of the PPNR-4-protein complex system by MD simulation. The protein secondary structures of PARP1 and NRP1 are shown in Figure 6A, B. It can be concluded from the figure that the secondary structures of the PARP1 and NRP1 proteins were stable during the MD simulations. The root-mean-squared deviation (rmsd) values of all atoms of PPNR-4 are plotted in Figure 6C, D, respectively. For PARP1, the rmsd values for PPNR-4 stabilized after 25 ns and fluctuated extremely small, with rmsd values roughly less than 0.15 nm. For NRP1, the rmsd values were constantly stable at 0.35 nm. The root-mean-square-fluctuation (rmsf) values of PARP1 and NRP1 are depicted in Figure 6E, F. For PARP1, the rmsf values for the key residues (Ser904, Gly863, Arg878, Asp766, Glu763, Tyr907, Tyr889, and Tyr896) were all less than 0.2 nm. For NRP1, the rmsf values for the key residues (Asp48, Ile143, Lys79, Tyr81, and Thr77) were all less than 0.15 nm. These results suggest the stable binding of PPNR-4 to the protein. In addition, Figure 6G, H show the radius of gyration (Rg) of the PARP1 and NRP1. The Rg values of both PARP1 and NRP1 fluctuated less than 0.2 nm, indicating that the proteins maintained their structural tightness during the simulation process.

The total binding free energy was calculated by MMPBSA method. The results are shown in Table 6. The total binding free energies for the PARP1-PPNR-4 complex and NRP1-PPNR-4 complex were −82.77 ± 9.67 kcal/mol and −17.82 ± 2.45 kcal/mol, respectively. For both complexes, the total gas-phase free energy and electrostatic energy were the main binding forces. In addition, van der Waals energy also contributed to the stability of the binding.

**TABLE 6:** Predicted free energies of PPNR-4 binding to PARP1 or NRP1.

Energy terms (kcal/mol)	PARP1	NRP1
van der Waals energy	−65.83 ± 3.69	−27.88 ± 2.72
Electrostatic energy	−90.57 ± 12.37	−22.74 ± 7.21
Polar solvation energy	82.15 ± 5.89	36.45 ± 7.35
Nonpolar solvation energy	−8.52 ± 0.20	−3.65 ± 0.41
Total gas-phase free energy	−156.40 ± 12.28	−50.62 ± 8.15
Total solvation free energy	73.63 ± 5.84	32.80 ± 7.16
Total binding free energy	−82.77 ± 9.67	−17.82 ± 2.45

### 3.6 Cell growth inhibitory activity

**FIGURE 7:**
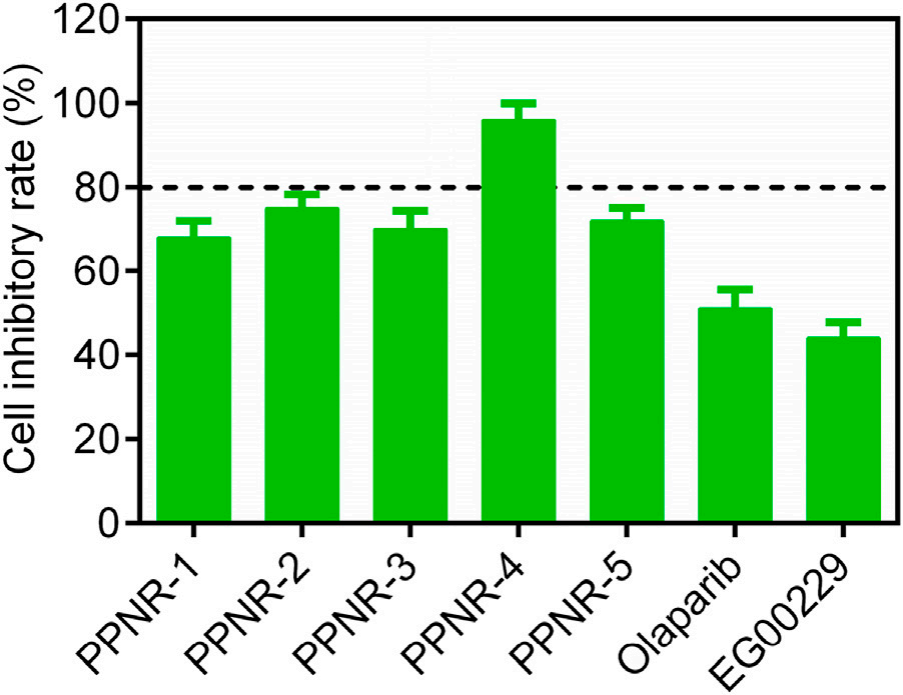
The cell inhibitory rate (%) of PPNR 1-5 against MDA-MB-231 cells at a concentration of 2 μM. Olaparib and, EG00229 are used as a positive control. Data are presented as the mean ± SD (n = 3).

Since PARP1 and NRP1 play important roles in the development of breast cancer, we further investigated the cellular antiproliferative activity of PPNR 1–5. PARP1 and NRP1 are expressed upregulated in MDA-MB-231 cells, whereas their expression is low in normal human mammary epithelial cells (MCF-10A) (Lee et al., 2009; Arun et al., 2015). As seen in Table 7, at a concentration of 2 μM, the inhibition rate of PPNR-4 on MDA-MB-231 cells was higher than 80%. After 7 days cell treatment with PPNR 1-5, antiproliferative activity of PPNR 1-5 varied from 0.21 μM to 0.89 μM. The activity of PPNR-4 (IC_50_ = 0.21 μM) on MDA-MB-231 cells was significantly stronger than that of olaparib (IC_50_ = 0.96 μM) and, EG00229 (IC_50_ = 62.45 μM) (Figure 7; Supplementary Figure S3). We investigated the selectivity of PPNR-4 towards normal human mammary epithelial cells. The results are shown in Table 8. The inhibitory activity of PPNR-4 on MDA-MB-231 cells was much higher than that on normal human mammary epithelial cells MCF-10A (IC_50_ > 10 μM). In addition, we tested comprehensive toxicity of PPNR-4 across a range of normal cell types. PPNR-4 showed no inhibitory effects (IC_50_ > 10 μM) on normal human cells (L02, FHC, PNT1A, HEL-299, and HEK-293), proving the safety of PPNR-4 on a wider range of normal cell types (Table 8).

**TABLE 7:** The cytotoxicity of PPNR 1-5 on MDA-MB-231 cell line.

Compounds	IC_50_ (μM)^a^
PPNR-1	0.89
PPNR-2	0.56
PPNR-3	0.83
PPNR-4	0.21
PPNR-5	0.72
Olaparib	0.96
EG00229	62.45

^a^ IC_50_ (μM) is the concentration of compound needed to reduce cell growth by 50% following 7 days cell treatment with PPNR, 1-5.

**TABLE 8:** The cytotoxicity of PPNR-4 on normal human cells.

Cells	IC_50_ (μM)
MCF-10A	>10
L02	>10
FHC	>10
PNT1A	>10
HEL-299	>10
HEK-293	>10

Overall, these results indicate that PPNR-4 showed highly potent inhibitory activity *in vitro* against multiple types of breast cancer cells, with a significantly higher inhibition rate than positive controls.

### 3.7 Functional effects of PPNR-4 on PARP1 and NRP1

**FIGURE 8:**
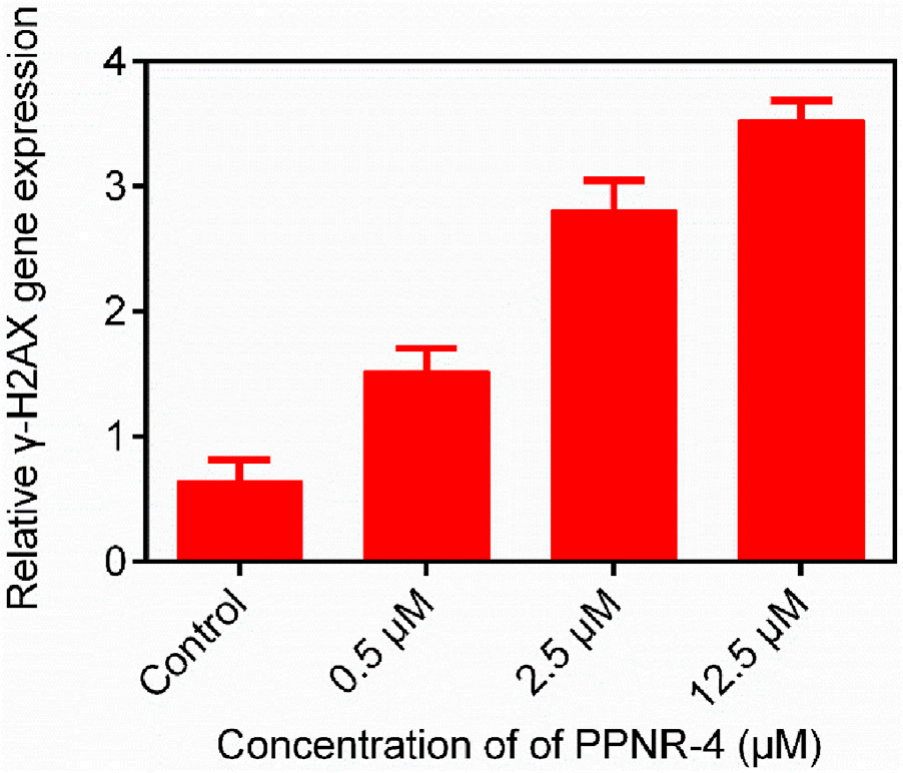
The γ-H2AX gene expression after 24 h of PPNR-4 treatment on KG-1 cells. Data are presented as the mean ± SD (n = 3).

We investigated the functional effects of PPNR-4 on PARP1 and NRP1. Since γ-H2AX is a molecular marker break in double-stranded DNA, it is regarded as a parameter of PARP1 (Bonner et al., 2008). Therefore, we investigated whether PPNR-4 increased γ-H2AX levels. Figure 8 shows the γ-H2AX levels at 0.5 μM, 2.5 μM and 12.5 μM after treatment with PPNR-4. These results indicate that PPNR-4 interfered with DNA repair function through inhibition of PARP1 and led to DNA double-strand breaks.

**FIGURE 9:**
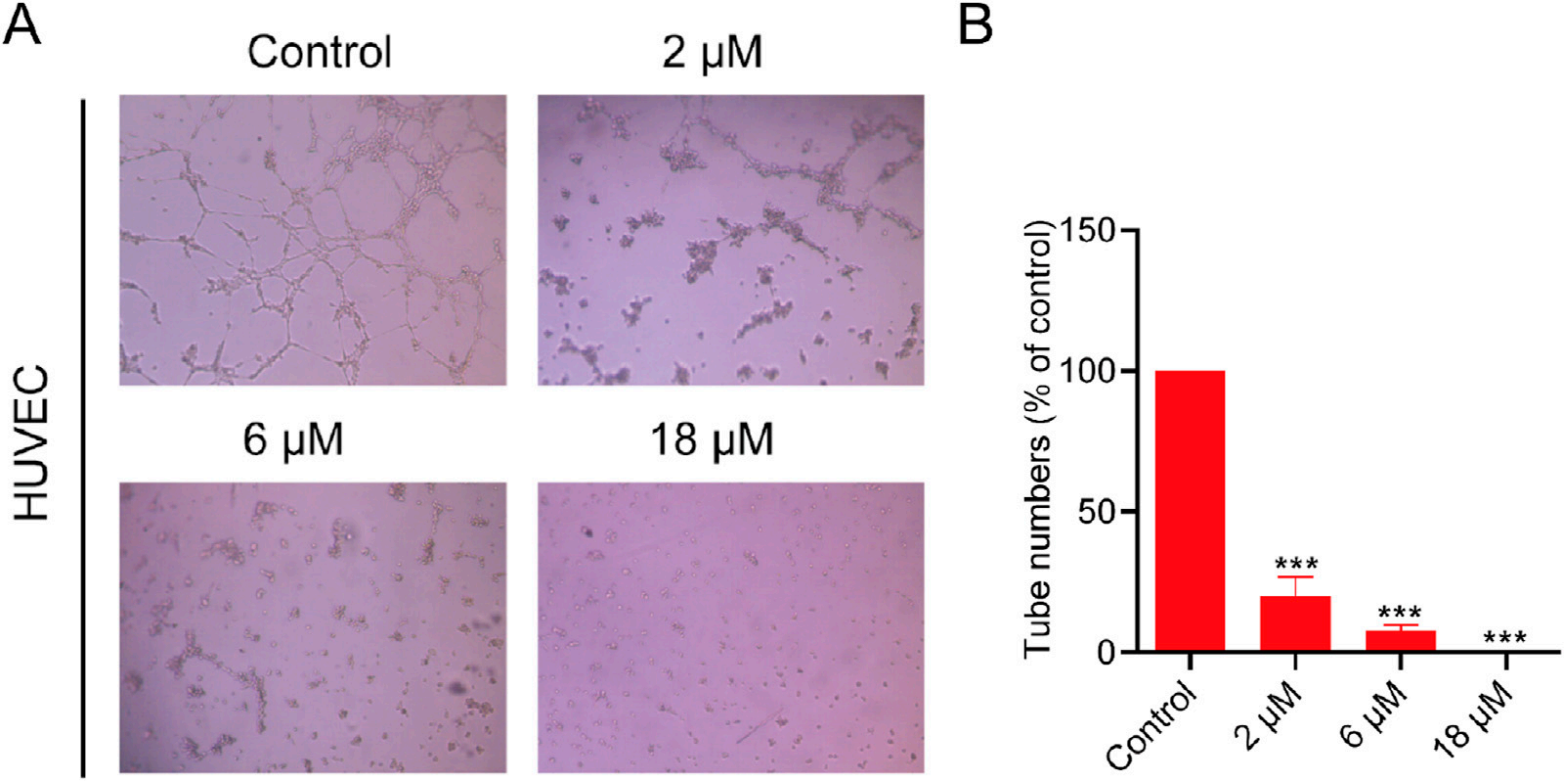
The effects of PPNR-4 on the tube formation capacity of HUVECs. **(A)** The tube formation capacity was measured in HUVECs treated with PPNR-4 (0, 2, 6, and 18 μM). **(B)** The results of **(A)** were quantified. Data are presented as the mean ± SD (n = 3). ***p < 0.001 vs control.

It is well-known that NRP1 plays an essential role in angiogenesis. The tube formation of human umbilical vein endothelial cells (HUVECs) represents the critical step in angiogenesis (Plein et al., 2014). We used HUVECs as a model to examine the angiogenic properties of PPNR-4. As shown in the Figure 9, PPNR-4 significantly interfered with tube formation in a dose-dependent manner compared with the control group. In addition, we measured the viability of HUVEC cells in the same experimental conditions. PPNR-4 inhibited HUVEC cell proliferation (Supplementary Figure S4). Thus, these results suggest that PPNR-4 significantly inhibited angiogenesis.

### 3.8 *In vivo* pharmacokinetic (PK) profile

We further conducted an *in vivo* pharmacokinetic (PK) study of PPNR-4. PPNR-4 was administered orally (p.o) at a dosage of 10 mg/kg in male Sprague-Dawley rats, and the main pharmacokinetic parameters are listed in Table 9. It demonstrated good oral drug exposure (AUC = 8,023 ng h/mL) with oral bioavailability (*F*) of 66.5%, suggesting that oral administration would be a suitable dosing route for further pharmacodynamic study. The terminal half-life (T_1/2_) was 2.71 h and the maximum plasma concentration (C_max_) was 812 μg/mL.

**TABLE 9:** Pharmacokinetic profile for PPNR-4 in SD rats^a^.

Compound	Route	Dose (mg/kg)	T_1/2_ (h)	C_max_ (μg/mL)	AUC (ng·h/mL)	*F* (%)
PPNR-4	p.o	10	2.71	812	8,023	66.5

^a^ T_1/2_, elimination half-life; C_max_, maximum plasma concentration; AUC, area under the drug−time curve; *F,* oral bioavailability.

### 3.9 *In vivo* antitumor effects

**FIGURE 10:**
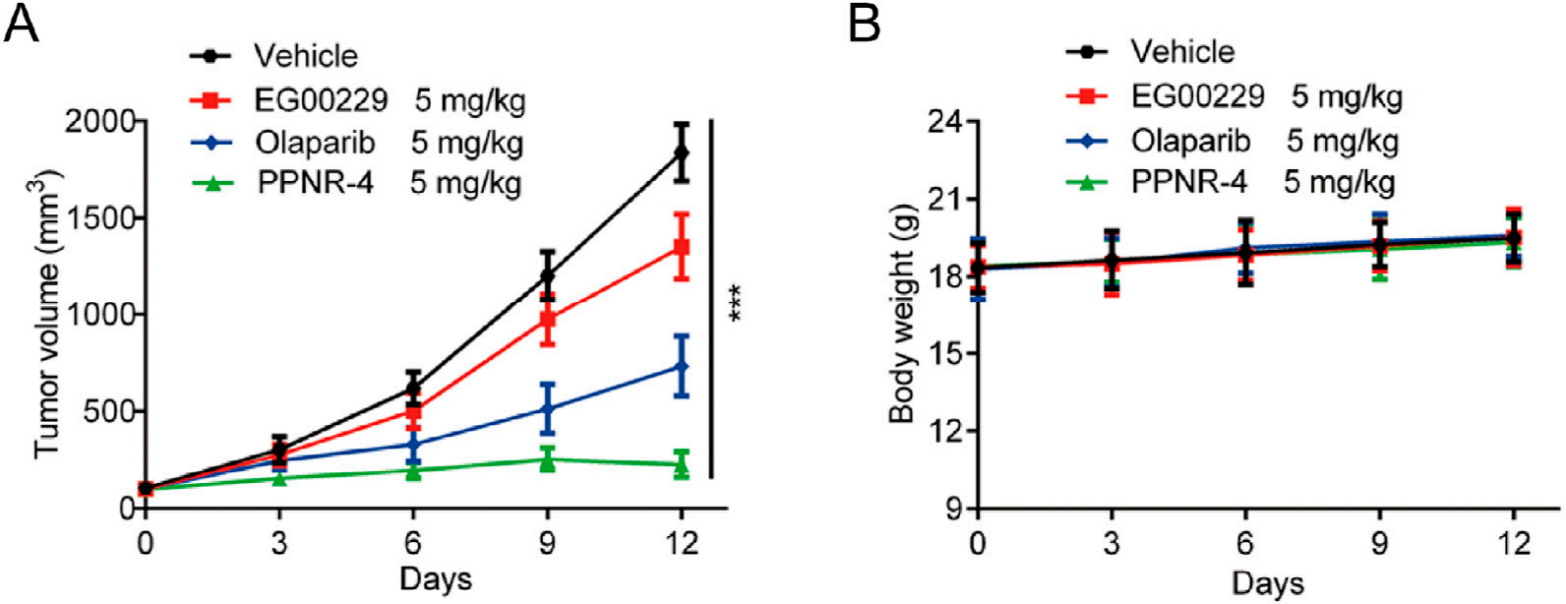
**(A)** *In vivo* antitumor effect of PPNR-4. Nude mice bearing tumor were randomly divided into four groups, and treated with PPNR-4 (5 mg/kg), EG00229 (5 mg/kg), olaparib (5 mg/kg) or vehicle (negative control), respectively. Tumor volumes were measured and calculated once every 3 days. **(B)** The change of body weight in mice. Data are presented as the mean ± SD, n = 6. ***p < 0.001 means a significant difference *versus* the vehicle group.

PPNR-4 displayed excellent antitumor activity *in vitro* assays. Thus, a mouse MDA-MB-231 xenograft tumor model was established to assess the *in vivo* antitumor activity of PPNR-4. Nude mice bearing tumor were randomly divided into four groups, and treated with PPNR-4 (5 mg/kg), olaparib (5 mg/kg), EG00229 (5 mg/kg), and vehicle. It was clear that the PPNR-4-treated group had a more remarkable inhibitory effect on tumor volume growth in mice in comparison to the olaparib or, EG00229-treated groups (Figure 10A). The body weight data of the mice are shown in Figure 10B. The body weights of mice showed no apparent change in all groups. As a result, PPNR-4 had significant antitumor effects *in vivo* at a dose of 5 mg/kg.

## 4 Conclusion

Nowadays, the number of newly diagnosed cases of breast cancer is rapidly increasing every year. Given the important role of PARP1 in human breast cancer and the issue of drug resistance to PARP1 inhibitors, the development of novel and efficient anticancer agents is an important way to prevent cancer proliferation and migration. Thus, finding more potent dual inhibitors based on novel dual targets is still in high demand. In this study, we successfully identified several new dual-targeted PARP1/NRP1 inhibitors through structure-based virtual screening. Docking studies revealed the possible conformation of PPNR-4 binding to the PARP1 and NRP1. PPNR-4 exhibited robust potency, inhibiting PARP1 (IC_50_ = 7.71 ± 0.39 nM) and NRP1 *in vitro*. In addition, the cellular assays verified that PPNR-4 possessed the most excellent antiproliferative activity (IC_50_ = 0.21 μM). Molecular dynamics (MD) simulations also demonstrated that PPNR-4 could stably bind to receptor proteins PARP1 and NRP1. Furthermore, PPNR-4 demonstrated excellent *in vivo* antitumor efficacy. Most notably, we found that the results of the cytotoxicity experiments closely matched the results of the docking score. This confirmed the plausibility of the predictions of our virtual screening approach. In this study, we mainly focused on the discovery of a potent anticancer drug (PPNR-4). Further exploring the resistance of cancer cells to PPNR-4 is a good direction for future research. In the future, we will continue to conduct long-term studies on PPNR-4 and determine its potential resistance mechanism. Moreover, we will further evaluate the efficacy of PPNR-4 in combination with other cancer therapies in future studies to explore potential synergistic effects and broaden its therapeutic applicability.

## Data Availability

The original contributions presented in the study are included in the article/Supplementary Material, further inquiries can be directed to the corresponding author.
