# Supplementary material for: Discovery of novel PARP1/NRP1 dual-targeting inhibitors with strong antitumor potency
Source: Front Pharmacol. 2024 Nov 29;15:1454957. doi: 10.3389/fphar.2024.1454957 (PMC11637875; doi:10.3389/fphar.2024.1454957)
Supplement: Supplementary file 1 [file DataSheet1.docx]

**Supplementary material**

**Discovery of novel PARP1/NRP1 dual-targeting inhibitors with strong antitumor potency**

**Juanjuan Liu^†,1^, Yifei Geng^†,2^, Su Jiang^†,1^ Lixia Guan^2^, Junyi Gao^1^, Miao-Miao Niu^2^, and Jindong Li^*,1^**

^1^Department of Pharmacy, Taizhou School of Clinical Medicine, The Affiliated Taizhou People’s Hospital of Nanjing Medical University, Taizhou 225300, China

^2^Department of Pharmaceutical Analysis, China Pharmaceutical University, Nanjing 211198, China

†These authors contributed equally to this work.

*** Correspondence:**Corresponding Author
lijindong20230419@njmu.edu.cn (Jindong Li)

**

**

**Figure S1.** Concentration-response curves for IC_50_ values of PPNR-4 inhibition of PARP1(A) and NRP1 (B).

**
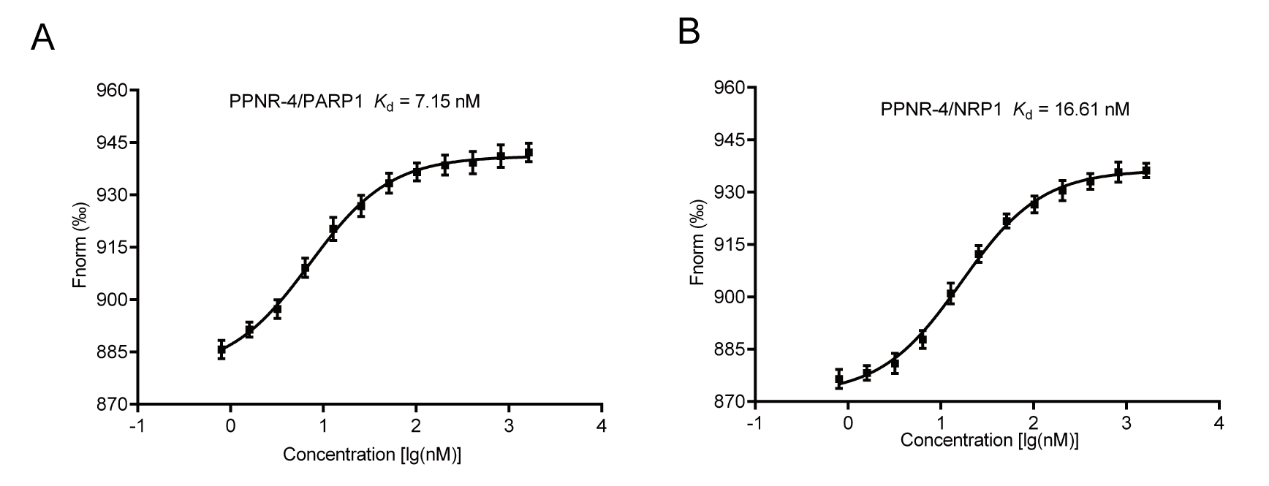
**

**Figure S2.** Concentration-response curves of *K*_d_ values of PPNR-4 to PARP1 (A) and NRP1 (B) by MST assay. Data are presented as the mean ± SD (n = 3).

**
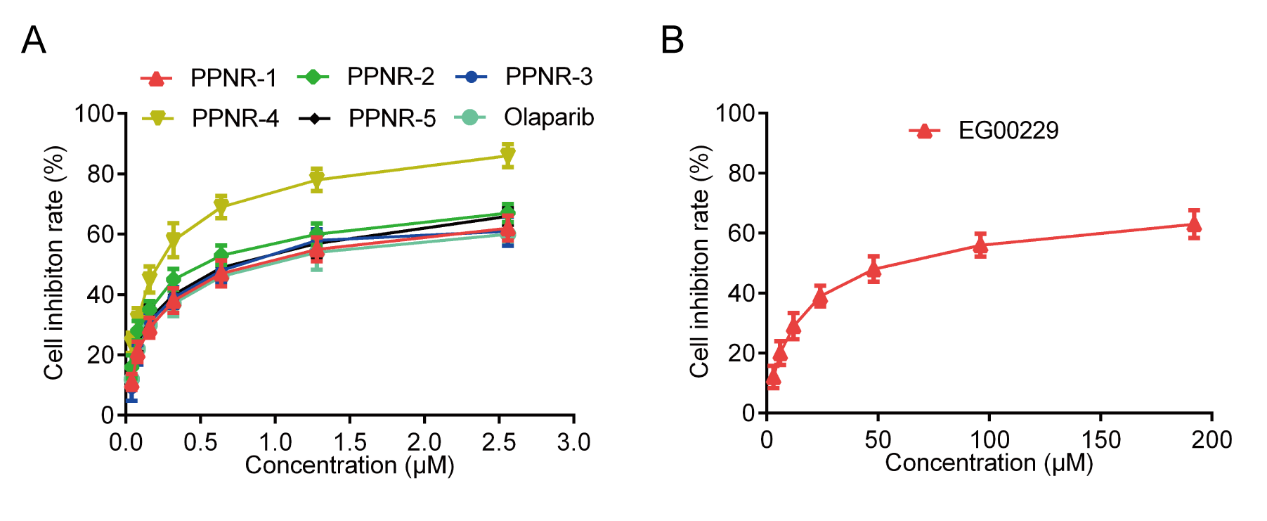
**

**Figure S3.** (A) The cytotoxicity of PPNR 1-5 and olaparib on MDA-MB-231 cell line. (B) The cytotoxicity of EG00229 on MDA-MB-231 cell line. Data are presented as the mean ± SD (n = 3).

**

**

**Figure S4.** The cytotoxicity of PPNR-4 on HUVEC cells. Data are presented as the mean ± SD (n = 3).

**Table S1.** Relative number of tube formations (% of control).

| **Control** | **PPNR-4** | | |
| --- | --- | --- | --- |
|  | **2 μM** | **6 μM** | **18 μM** |
| 100 | 14.00 | 6.00 | 0 |
| 100 | 15.69 | 5.88 | 0 |
| 100 | 21.15 | 9.62 | 0 |
| 100 | 29.09 | 9.09 | 0 |

**Table S2.** The raw data of tumor volume (mm^3^).

| **Days** | **Vehicle** | | | **EG00229** | | | **Olaparib** | | | **PPNR-4** | | |
| --- | --- | --- | --- | --- | --- | --- | --- | --- | --- | --- | --- | --- |
|  | Mean | SD | n | Mean | SD | n | Mean | SD | n | Mean | SD | n |
| 0 | 106 | 33 | 6 | 101 | 31 | 6 | 103 | 35 | 6 | 99 | 29 | 6 |
| 3 | 302 | 67 | 6 | 275 | 54 | 6 | 245 | 48 | 6 | 155 | 34 | 6 |
| 6 | 619 | 83 | 6 | 502 | 92 | 6 | 329 | 86 | 6 | 196 | 41 | 6 |
| 9 | 1203 | 124 | 6 | 976 | 131 | 6 | 513 | 126 | 6 | 253 | 57 | 6 |
| 12 | 1837 | 145 | 6 | 1352 | 167 | 6 | 733 | 154 | 6 | 226 | 65 | 6 |

**Table S3.** The changes of body weight (g) in mice.

| **Days** | **Vehicle** | | | **EG00229** | | | **Olaparib** | | | **PPNR-4** | | |
| --- | --- | --- | --- | --- | --- | --- | --- | --- | --- | --- | --- | --- |
|  | Mean | SD | n | Mean | SD | n | Mean | SD | n | Mean | SD | n |
| 0 | 18.34 | 0.96 | 6 | 18.38 | 0.84 | 6 | 18.29 | 1.17 | 6 | 18.41 | 0.92 | 6 |
| 3 | 18.65 | 1.10 | 6 | 18.51 | 1.22 | 6 | 18.55 | 0.94 | 6 | 18.62 | 0.84 | 6 |
| 6 | 18.93 | 1.24 | 6 | 18.84 | 0.97 | 6 | 19.12 | 0.99 | 6 | 18.85 | 1.09 | 6 |
| 9 | 19.24 | 0.86 | 6 | 19.19 | 0.95 | 6 | 19.37 | 1.03 | 6 | 19.07 | 1.18 | 6 |
| 12 | 19.49 | 0.92 | 6 | 19.52 | 1.09 | 6 | 19.59 | 0.81 | 6 | 19.33 | 0.95 | 6 |
